# The Role of Diet and Nutrition in Cancer: Prevention, Treatment, and Survival

**DOI:** 10.3390/nu14163329

**Published:** 2022-08-14

**Authors:** Hiroto Narimatsu, Yuri Tanaka Yaguchi

**Affiliations:** 1 Cancer Prevention and Cancer Control Division, Kanagawa Cancer Center Research Institute 2-3-2 Nakao, Asahi-ku, Yokohama 241-8515, Japan; 2 Graduate School of Health Innovation, Kanagawa University of Human Services, Kawasaki 210-0821, Japan; 3 Department of Genetic Medicine, Kanagawa Cancer Center, 2-3-2 Nakao, Asahi-ku, Yokohama 241-8515, Japan; 4 Faculty of Education, Art, and Sciences, Yamagata University, 1-4-12 Kojirakawa-machi, Yamagata 990-8560, Japan

Diet and nutrition are important factors in cancer prevention and treatment because an unbalanced diet increases the risk of cancer onset, while malnutrition negatively impacts the efficacy of cancer treatment. Recent epidemiologic research has identified lifestyle and genetic factors associated with cancer prevention, while the identification of novel molecular agents has drastically changed treatment strategies. Since outcomes have improved for cancer patients, management of survivors has received increasing attention, especially concerning the role of diet and nutrition.

Research on cancer and nutrition to date has focused primarily on prevention, and there remains little research on treatment and care. In this context, research investigating the relationship between nutrition and cancer treatment is valuable. In this Special Issue, some promising insights on the roles of diet and nutrition in cancer prevention [1,2], treatment [3,4,5,6], and survivorship [7,8] are reported; diet and nutrition have a potentially significant clinical impact. Two studies on triple-negative breast cancers provide novel approaches to this cancer subtype [3,4]. Triple-negative breast cancer often has an unfavorable outcome because targeted therapies including hormone or *HER*-2 targeted therapy are not effective. Hossain et al. and Coleman et al. focused on the mycobiome and folate, respectively. Although these are preclinical studies, they provide us with novel approaches to improve outcomes in patients with this subtype of breast cancer, in which the use of chemotherapy and molecular agents is limited.

One study of this Special Issue indicated that a balanced diet is important in preventing cancers, with diet quality reported to reduce the risk of lung cancer [2]. Although isoflavones are associated with a reduced risk of breast cancer, a systematic review by Finkeldey, which included 18 RCTs with pre- and postmenopausal women, failed to present a clear association [9]. Further studies are needed on this topic. In terms of gene–environment interactions, particularly a study plan investigating gene-diet interaction is promising. If an interaction between genes and isoflavones is shown to have a high impact on cancer onset, considering this interaction may help in establishing a clear association between isoflavones and cancers [1]. Prospective large cohort studies investigating these interactions are ongoing worldwide [10,11]. Their findings from such cohort studies may provide additional valuable insights in this topic.

Cancer patients and survivors need appropriate nutrition support in addition to surgical or pharmacological treatment. This may improve not only the quality of life but also treatment outcomes, even for cancers with an unfavorable prognosis such as pancreatic cancer [12]. We believe that further studies are necessary for research on nutrition support for cancer patients. To increase the effectiveness of treatment in cancer patients in the future, we hope to see more research on nutritional care to prevent undernutrition and loss of quality of life.
